# The association of genetic polymorphisms with neuroconnectivity in breast cancer patients

**DOI:** 10.1038/s41598-021-85768-4

**Published:** 2021-03-17

**Authors:** Rebecca A. Harrison, Vikram Rao, Shelli R. Kesler

**Affiliations:** 1 Department of Neuro-Oncology, University of Texas MD Anderson Cancer Center, 1515 Holcombe Blvd, Unit 431, Houston, TX 77030 USA; 2 Cancer Neuroscience Laboratory, School of Nursing, University of Texas at Austin, Austin, TX 78712 USA; 3 Department of Diagnostic Medicine, Dell School of Medicine, University of Texas at Austin, Austin, TX 78712 USA; 4 LIVESTRONG Cancer Institutes, Dell School of Medicine, University of Texas at Austin, Austin, TX 78712 USA

**Keywords:** Cancer, Neuroscience, Psychology, Neurology, Oncology

## Abstract

Genetic polymorphisms in select genes, including *APOE* (apolipoprotein E), *COMT* (Catechol-O-Methyltransferase), *MDR1* (multi-drug resistance 1), *BDNF* (brain derived neurotrophic factor), and *GST* (glutathione-S-transferase), have been associated with vulnerability to cognitive impairment. In this study, we evaluated the relationship of these genetic variants to measures of brain health in patients with breast cancer, including neurocognitive testing and functional connectome analysis. Women with breast cancer (n = 83) and female healthy controls (n = 53) were evaluated. They underwent resting-state functional MRI scans and neurocognitive testing. Polymerase chain reaction (PCR) was performed on saliva samples to identify single nucleotide polymorphisms (SNPs) in candidate genes: *APOE, COMT, MDR1, BDNF,* and *GST*. Breast cancer patients treated with chemotherapy had slower processing speed (*p* = 0.04) and poorer reported executive function (*p* < 0.0001) than healthy controls. Those chemotherapy-treated patients that were *APOE e4* carriers had significantly slower processing speed. A greater number of risk-related alleles was associated with poorer connectivity in the regions of the left cuneus and left calcarine. While breast cancer patients that are *APOE e4* carriers may have a select vulnerability to processing speed impairments, other risk-related alleles were not found to influence cognitive test performance in this population. Conversely, regions of impaired functional connectivity appeared to be related to risk-related genetic polymorphisms in breast cancer patients. This suggests that a cancer patient’s SNPs in candidate genes may influence the risk of neurotoxicity. Further study evaluating the impact of genotype on biomarkers of brain health in cancer survivors is warranted.

## Introduction

With an increasing cancer incidence and decreasing death rate^1^, the public health impact of cancer survivorship is of central importance. In addition to the direct sequelae of a malignancy itself, cancer has an established impact on cognitive function^2,3^. Cancer patients have a 40% greater likelihood of reporting memory complaints than those without cancer, affecting those with active cancer and those in remission^4^. There are often multiple contributors to cancer related cognitive impairment (CRCI), with surgery, chemotherapy, radiation, psychosocial stress, and the cancer itself all having demonstrated contributions^5^. The biologic underpinnings of CRCI are likely complex, with both preclinical work and human neuroimaging studies have played central roles in elucidating some of these underlying mechanisms^6^. Mechanisms, including oxidative stress, mitotic inhibition of neural and glial progenitor cells, and induction of neural apoptosis have supportive contributory data^7^, and accelerated cortical ageing is now a central biologic construct in CRCI^8^.

More recently, the concept of germline genetic differences imparting increased vulnerability to CRCI has been explored, with genes associated with neurodegenerative diseases and enhanced toxicity from neurologic insults being implicated^9–12^. Carriers of the epsilon 4 allele of apolipoprotein E (*APOE*), associated with impaired neuronal membrane repair and synaptic plasticity^13^, has been associated with increased incidence of cognitive impairment after chemotherapy exposure^9,14^. Similarly, polymorphisms of other genes associated with neuronal and glial health and resilience, including *COMT* (Catechol-O-Methyltransferase), *MDR1* (multi-drug resistance 1), *BDNF* (brain derived neurotrophic factor), and *GST* (glutathione-S-transferase), also have a proposed role in augmenting neurotoxicity^11,15–17^.

Neuroimaging studies have demonstrated changes in brain structure and function in cancer patients, lending insight into the pathophysiology of cognitive impairment in these patients^6^. Connectome analysis is the mathematical representation on the brain’s functional and structural networks. It provides insight into brain circuitry, including the integration and segregation of networks^18–21^. Functional connectome organization in this cohort of chemotherapy-treated breast cancer survivors compared to healthy female controls was previously evaluated, with decreased clustering as well as alterations of nodal degree in frontal, striatal and temporal areas being observed^22^. Here we expand upon those results by including a chemotherapy-naïve breast cancer comparison and exploring genetic contributions to chemotherapy-related connectome injury and cognitive deficit. We also focused on evaluation of regional effects using network-based analysis which represents an improvement over our previous approach.

## Methods

### Participants

For this retrospective study, we used all available cases from our laboratory for breast cancer survivors who had neuroimaging, neuropsychological and genetic data. This included 83 women age 41–74 years with a history of primary breast cancer (stage I-IIIA) who had completed their primary treatment (surgery, chemotherapy and/or locoregional radiation therapy) more than 6 months (mean = 5.5 + /- 5.2 years; range = 0.7–28 years) prior to study entry and were currently without evidence of active cancer or gross neuropathology. They were compared with 53 healthy female controls (Table 1). In the breast cancer group, 42 women had a history of chemotherapy treatment while the remaining 41 were chemotherapy naïve. Individual chemotherapy treatment protocols included doxorubicin/cyclophosphamde/paclitaxel or docetaxel = 16, cyclophosphamide/methotrexate/5-fluorouracil = 4, doxorubicin/cyclophosphamide = 11, cyclophosphamide/paclitaxel or docetaxel = 9, doxorubicin/cyclophosphamide/5-fluorouracil = 1 and epirubicin/ cyclophosphamide/paclitaxel = 1. Additional treatment information is provided in Table 1. The Stanford University Institutional Review Board approved this study which was conducted according to the principles expressed in the Declaration of Helsinki. All participants provided written informed consent.

**Table 1:** Demographic, genotype and clinical data show as mean (standard deviation) unless otherwise noted.

	Chemotherapy N = 42	Chemotherapy naive N = 41	Healthy control N = 53
Age	55 (7)	59 (7)*	55 (9)
Age range	43–73	41–74	41–71
Education (yr)	16 (3)	17 (2)	17 (3)
Breast radiation	73%	65%	
Endocrine therapy (tamoxifen)	43%	58%	
Disease stage at diagnosis (0, I, II, III)	0%, 26%, 55%, 19%	37%*, 50%*, 13%*, 0%*	
Time since primary treatment (yr)	4.8 (4.8)	6.5 (5.6)	
Postmenopausal	89%	82%	44%*
Clinical assessment of depression score	51 (11)*	44 (10)	44 (9)
APOE e4	31%	24%	23%
BDNF Met	43%	28%	36%
MDR1 T	81%	78%	83%
GST Null	38%	42%	51%
COMT Val	88%	91%	95%

*Denotes significant (*p* < 0.05) difference.

Breast cancer survivors were excluded for history of relapse or prior anti-cancer treatment. All participants were excluded for diagnosed psychiatric, neurologic or comorbid medical conditions that are known to affect cognitive function as well as pregnancy, MRI contraindications or major sensory deficits (e.g. blindness)^23^. Breast cancer and comparison participants were recruited via the Army of Women (http://www.armyofwomen.org/), community flyer postings and advertisements. Breast cancer participants were also recruited via local support groups and physician referrals from the Stanford Women’s Cancer Center.

### Neurocognitive function testing

Subtests from the Wechsler Adult Intelligence Scales Fourth Edition^24^ were used to measure processing speed (Symbol Search) and working memory (Digit Span). Executive function was assessed using the Neuropsychological Assessment Battery Categories test^25^ and verbal fluency was measured using the Delis-Kaplan Executive Function System Letter Fluency subtest^26^. Verbal memory was assessed using the Hopkins Verbal Learning Test Revised Version^27^, which assesses both immediate and delayed recall. We also administered the Behavioral Rating Inventory of Executive Function (BRIEF), a patient reported measure of executive function^28^ and the Clinical Assessment of Depression (CAD) which measures depression, anxiety and fatigue^29^. Test scores were converted to T scores (mean = 50 + /- 10) based on the published normative data for each test. Some participants had missing data for certain assessments.

### MRI acquisitions

A subset of participants underwent MRI scanning which was performed on a GE Discovery MR750 3.0 T whole body scanner (GE Medical Systems, Milwaukee, WI). Functional MRI (fMRI) data were acquired while participants rested in the scanner with their eyes closed using a T2* weighted gradient echo spiral pulse sequence: relaxation time = 2000 ms, echo time = 30 ms, flip angle = 80° and 1 interleave, field of view = 220, matrix = 64 × 64, in-plane resolution = 3.125. Number of data frames collected was 216, thus total scan time was 7:12. An automated high-order shimming method based on spiral acquisitions was employed to reduce field heterogeneity^30^. To coregister and normalize functional images with a standardized template, a high-resolution, 3 dimension inversion-recovery prepared fast spoiled gradient echo anatomical scan was acquired: relaxation time: minimum, echo time: minimum, flip: 11 degrees, inversion time: 300 ms, bandwidth: ± 31.25 kHz, field of view: 24 cm, phase field of view: 0.75, slice thickness: 1.5 mm, 125 slices, 256 × 256 at 1 excitation, scan time: 4:26. Two task based fMRI scans and a diffusion weighted scan were also acquired during the MRI session that are utilized in other analyses not reported here. However, the resting state scan was acquired prior to any other scans to reduce the effects of specific cognitive processes on the resting state networks.

### Functional MRI preprocessing

Image preprocessing was performed using Statistical Parametric Mapping 8 (Wellcome Trust Centre, London, UK). Briefly, images were realigned to correct for head motion, segmented and coregistered with the anatomic MRI, normalized to a standard space (MNI) template and smoothed with an 8 mm FWHM kernel. We defined 90 cortical and subcortical regions of interest (ROIs) using the Automated Anatomical Labeling atlas^31^ which were used to extract regional time series data. This was performed using the CONN Toolbox^32^. First, data were band pass filtered to 0.008 Hz—0.09 Hz. Then, the CompCor method^33^ was used to reduce physiological and other non-neuronal artifacts. This method involves extracting signal from white matter and cerebrospinal fluid regions using principal component analysis and then regressing these signals out of the total fMRI signal. Finally, temporal correlations between all possible pairs of regions were computed based on the corrected fMRI signal and normalized to z-scores, resulting in a 90 × 90 functional connectivity matrix for each participant. Negative functional edges were zeroed given evidence that properties of negative correlation networks are different than those of positive correlation networks^34,35^.

### Genotyping

Saliva samples were obtained from all participants using the Oragene DNA OG-250 collection kit (DNA Genotek, Kanata, Ontario). Genotyping was accomplished by polymerase chain reaction (PCR) fragment length polymorphism analysis with restricted fragment length polymorphisms for *APOE, COMT, MDR1, BDNF, GST.*

### Statistical analysis

#### Neurocognitive function

Test scores were compared between groups using analysis of covariance controlling for age. Significant omnibus tests were supplemented with Tukey’s honestly significant difference test.

#### Connectome

Global connectome properties including clustering, path length, and small-worldness were calculated using graph theoretical analysis as described previously^36–39^. Briefly, a network is defined as a set nodes (regions) and their connections (edges). The clustering coefficient of a node is the proportion of a nodes neighbors that are also neighbors with each other and is a measure of network segregation. Path length describes the minimum number of edges that separate pairs of nodes and is an indication of network integration. Small-worldness is an organization characteristic of brain networks and other large-scale complex biological networks that differentiates them from random networks. Small-worldness was calculated as [CC/CC_R_]/[PL/PL_R_] where CC = clustering coefficient and PL = characteristic path length and CC_R_ and PL_R_ are the mean clustering coefficient and characteristic path length of 100 random benchmark networks^40^.

Thresholding connectomes is necessary for removing false positive edges and facilitating between group comparisons but can remove potentially valid information regarding differences in network topology^41,42^. Therefore, we compared connectome properties across multiple densities using the area under the curve (AUC)^43,44^. Connectomes were first corrected for age via linear regression conducted at every ROI^45^. We then measured connectome properties at each density from minimum connection density to the last density associated with a small-world organization^46,47^ up to a maximum density of 0.5^48^ and calculated the AUC across this entire range. AUCs were compared pairwise between groups using nonparametric permutation testing using 5000 iterations and two-tailed *p* values as in our previous studies^22,49^. Specifically, AUCs from randomized groups with the same number of participants as the original groups were calculated to create a permutation distribution of between-group differences. The actual between group difference was then placed in the corresponding permutation distribution and a two-tailed *p* value was calculated based on its percentile position^45^.

Regional connectome differences were assessed using the Network-Based Statistic^50^ with age as a covariate. Unlike our previous approach which examined connectivity for individual nodes, this method identifies connected substructures, or components, within the larger network^50^. Permutation testing with 5000 permutations was then used to determine group differences in components controlling for multiple comparisons using false discovery rate (FDR)^50^.

Neurocognitive test scores and connectome properties that showed between group differences were further compared between genotypes (e.g. presence vs. absence of *APOE* e4, *COMT* Val, *BDNF* Met, *MDR1* T and *GST* null variants) within each participant group using t-tests or Wilcoxon rank tests, as appropriate, based on data visualization.

## Results

### Neurocognitive function

As shown in Table 2, the chemotherapy group demonstrated lower processing speed as measured by the Symbol Search test (F = 3.2, *p* = 0.04). They also rated themselves as having significantly more difficulties with executive functions than the other two groups (F = 20.4, *p* < 0.0001). Although it differed between groups, CAD score was not significantly associated with any objective cognitive scores but was related to BRIEF score (r = 0.62, *p* < 0.0001). However, the group comparison remained significant even after adding CAD as a covariate (F = 12.0, *p* < 0.0001).

**Table 2:** Neurocognitive function data shown as mean (standard deviation).

	N	Chemotherapy	N	Chemotherapy naive	N	Healthy control	*p* value
Verbal fluency	42	55 (11)	41	58 (11)	53	60 (12)	0.09
HVLT-total	42	52 (8.8)	41	53 (7.6)	41	53 (10)	0.79
HVLT-delayed	42	53 (7.5)	41	53 (9.1)	41	52 (9.8)	0.99
NAB categories	42	52 (9.8)	41	54 (7.5)	39	53 (9.4)	0.66
Symbol search	29	59 (8.3)	29	64 (9.3)	35	60 (9.6)	0.04
Digit span	41	53 (8.3)	40	55 (9.2)	49	55 (9.7)	0.55
BRIEF	42	61 (11)	41	50 (9.7)	52	48 (8.4)	< .0001

### Connectome properties

**Figure 1:**
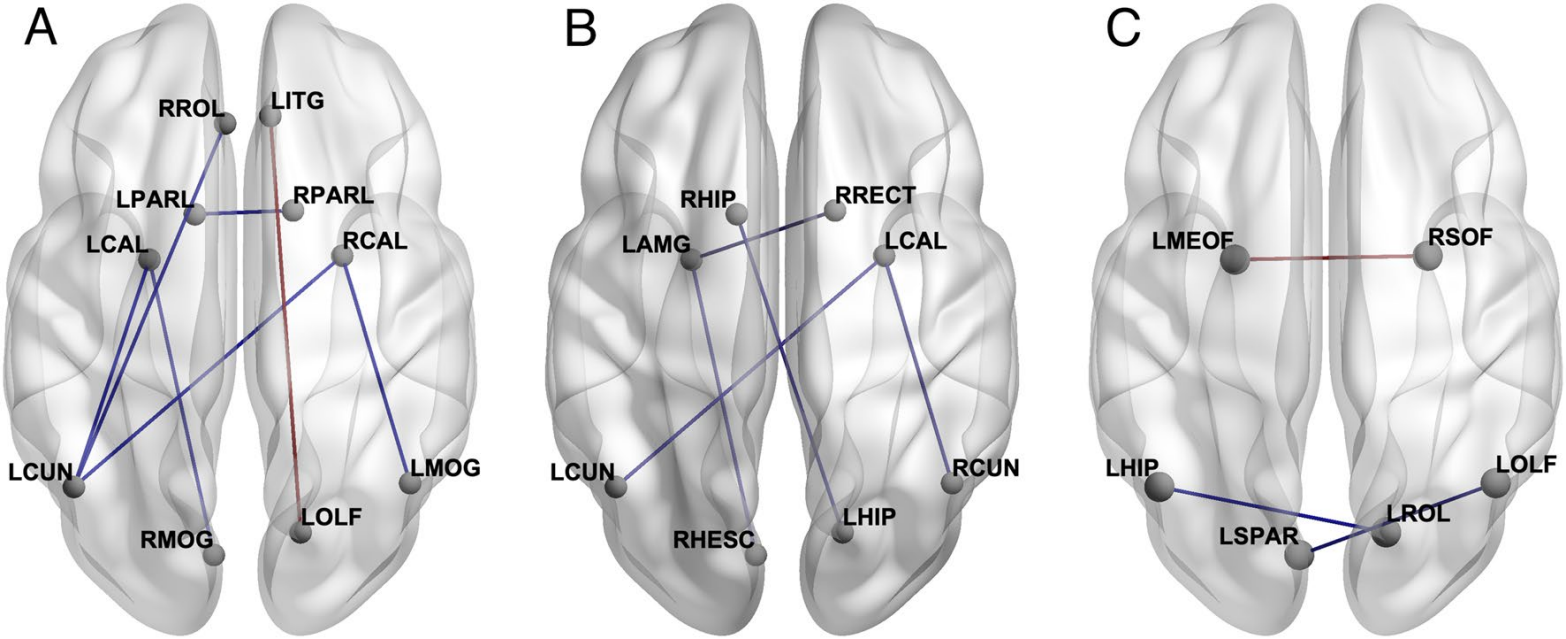
The chemotherapy group demonstrated significantly (*p* < 0.05, FDR corrected) hypo-connected (blue lines) and hyper-connected (red lines) edges compared to the chemotherapy naïve group (**A**) and hypo-connected edges compared to healthy controls (**B**). The chemotherapy naïve group showed both hypo-connected (blue lines) and hyper-connected (red lines) edges compared to healthy control (**C**). *LAMG* left amygdala, *LCAL* left calcarine, *LCUN* left cuneus, *LHIP* left hippocampus, *LITG* left inferior temporal gyrus, *LMEOF* left medial orbital frontal, *LMOG* left middle occipital gyrus, *LOLF* left olfactory, *LPARL* left parietal lobule, *LROL* left rolandic operculum, *LSPAR* left superior parietal lobe, *RCAL* right calcarine, *RCUN* right cuneus, *RHESC* right Heschl’s gyrus, *RHIP* right hippocampus, *RMOG* right middle occipital gyrus, *RPARL* right parietal lobule, *RRECT* right rectus gyrus, *RROL* right rolandic operculum, *RSOF* right superior orbital frontal. Figure created using BrainNet Viewer^51^.

The chemotherapy group demonstrated significantly lower clustering compared to the chemotherapy naïve group (*p* = 0.02). Additionally, they showed significantly lower characteristic path length (*p* = 0.04). The chemotherapy group showed hypo- and hyper-connectivity between several regions (Fig. 1).

Compared to healthy controls, the chemotherapy group demonstrated significantly lower clustering (*p* = 0.005) and characteristic path length (*p* = 0.02). Regionally, there were multiple areas of hypo-connectivity in the chemotherapy group (Fig. 1).

There were no differences in global connectome metrics between healthy controls and chemotherapy naïve survivors. However, there were some regions of hyper- and hypo-connectivity in the chemotherapy naïve group (Fig. 1). CAD score was not associated with connectome metrics (*p* > 0.52).

### Genotype and neurocognitive function

**Figure 2:**
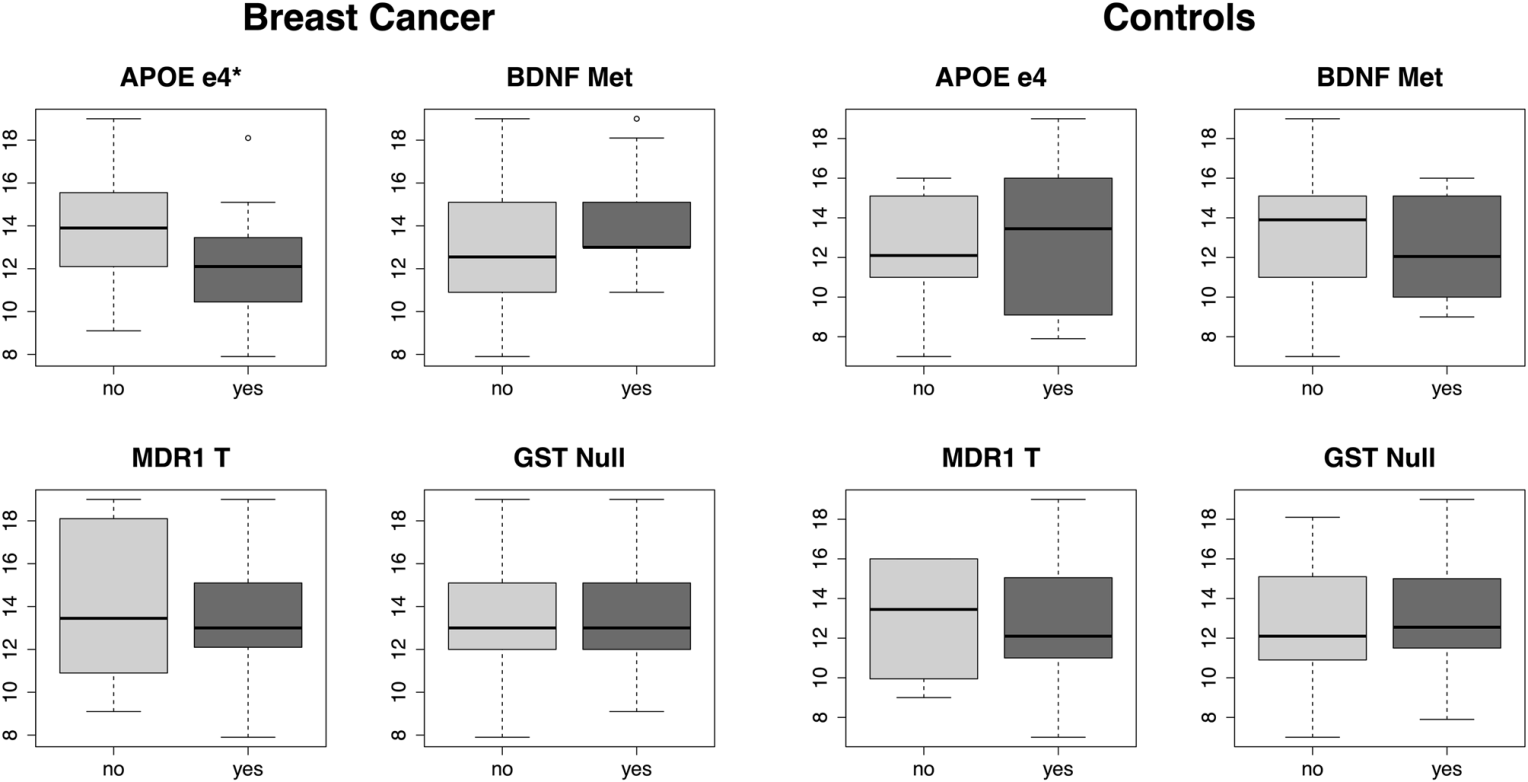
Processing speed performance by genotype. Symbol Search test score differed significantly only between *APOE* e4 groups. * *p* < 0.05.

Symbol Search (processing speed) performance was significantly lower in chemotherapy-treated survivors who had the *APOE* e4 allele (Fig. 2). No other comparisons were significant.

### Genotype and connectome function

**Figure 3:**
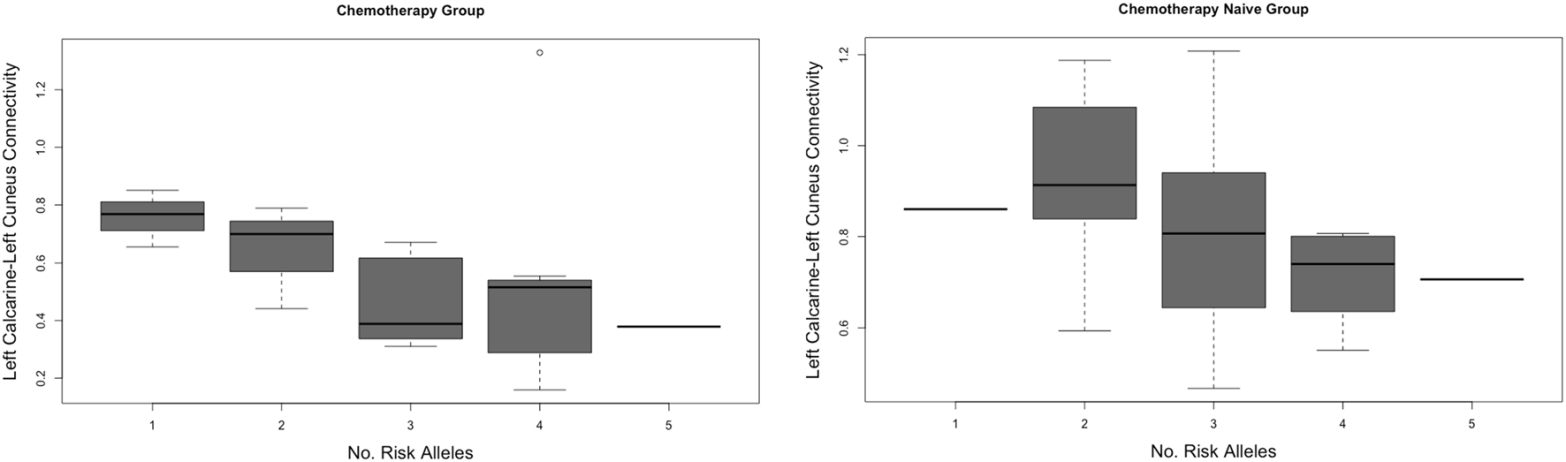
Correlation between left calcarine-left cuneus connectivity and number of risk-related alleles in the breast cancer groups.

There were no differences between genotype groups and global connectome properties. Regionally, connectivity between the left calcarine and left cuneus, which was lower in the chemotherapy group compared to both control groups, was significantly lower in chemotherapy-treated survivors who had the *BDNF* Met allele (W = 84, *p* = 0.04) as well as those who possessed the *MDR1* T allele (W = 49, *p* = 0.02). Additionally, the connectivity between right and left paracentral lobule, which was lower in the chemotherapy group compared to chemotherapy naïve group, was significantly lower in chemotherapy-treated survivors who had the *MDR1* T allele (W = 3, *p* = 0.01). Further, as shown in Fig. 3, lower left calcarine to left cuneus connectivity was significantly correlated with higher number of risk-related alleles in the chemotherapy group (r = −0.51, *p* = 0.02) and in the chemotherapy naïve group (r = −0.47, *p* = 0.01), but not in healthy controls (*p* = 0.20). In healthy controls who carried the *APOE* e4 allele, connectivity between left olfactory and left superior parietal lobe was higher (W = 30, *p* < 0.0001) while connectivity between left hippocampus and left rolandic operculum was lower (W = 199, *p* = 0.004). No other genotype comparisons or correlations were significant. There was not enough variance to conduct comparisons for the *COMT* genotype.

## Discussion

This innovative study evaluates the relationship of genetic polymorphisms associated with neural ageing and vulnerability to neurologic insults on cerebral connectivity. This is the first study to our knowledge where both cognitive function and the functional connectome, a demonstrated biomarker of CRCI^18,22,52^, have been evaluated in relation to these genetic signatures. With roles identified in neuronal repair and survival, dendritic and axonal growth, long term potentiation, CNS concentrations of cytotoxic therapies^16,53–57^, the genes evaluated in this study have an association with brain ageing, a proposed concept behind CRCI^8,58^. The functional connectome, representing key contributions of genetic, epigenetic, and environmental variables^59–61^, has been shown to be a viable biomarker of neurologic impact of cancer^18,52,62^. In this study, we identified that patients harboring those genetic signatures associated with accelerate cognitive ageing have associated alterations in brain functional connectivity, supporting genetic relationship to cancer-related neurotoxicity.

Of all cognitive domains evaluated on clinical testing, only processing speed had a significant interrelationship with genotype, with *APOE4* carriers showing impairment. The lack of association with other cognitive functions may be secondary to the low rates of cognitive impairment identified in this population as a whole. A lack of sensitivity of formal neuropsychological evaluation in corroborating subjective cognitive complaints has previously been identified^63^. The disparity between self-reported and objectively defined cognitive dysfunction is further supported in this study, with patients rating themselves as having significant cognitive issues illustrated by different BRIEF scores between the groups, despite the lack of executive dysfunction by clinical testing. The adult children of Alzheimer’s patients carrying the *APOE4* allele Alzheimer’s have been found to have slower processing speed than those that do not harbor this allele, and have lower white matter volumes^64^. Processing speed is a commonly affected domain in CRCI, and the *APOE4* allele could render this domain particularly vulnerable to dysfunction^58^.

A distinct global connectome organization was demonstrated in the chemotherapy group, including lower clustering, shorter characteristic path length, and hypo- and hyper-connectivity between multiple regions. These distinct alterations in large-scale brain networks have been highlighted previously in chemotherapy-treated patients^22^, highlighting the general impairments in information processing and network integration that can occur in cancer survivors. Conversely, no global differences were observed in connectome properties between genotype groups. There are several potential explanations for this finding. The selected at-risk alleles may not impact on global connectivity, instead leading to more focused regions of aberrant cerebral connectivity with relative preservation of global brain function. Furthermore, differences in the allele frequency in our test group may have led to these distinct results. While variable amongst ethnicities^65^, the prevalence of *APOE4* carriers in the general population is estimated at 14% globally^66^, a smaller proportion than in our study population. Similarly, the *MDR1* T allele has been identified in 53%^67^, but was higher in our evaluated population. As such, these could alter our identification of abnormalities.

The genetic polymorphisms evaluated in this study have may also predispose to cancer itself, adding further complexity to the interrelationship with brain health. *APOE* has been found to mediate tissue repair, immune response and regulation, and cell growth and differentiation^68^, biologic processes that influence individual susceptibility to malignancy. A meta-analysis evaluating the *APOE* gene in breast cancer patients identified one and a half times increased risk of breast cancer among Asians carrying the *e4* protein isoform of *APOE*, supporting this allele as a low-penetrant risk factor for development of breast cancer^69^. As the *COMT* gene is an estrogen-metabolizing enzyme, polymorphisms have been postulated to impact breast cancer risk, with catechol estrogens having proposed oncogenic influence. Despite this theoretical relationship, population-based studies have failed to demonstrate a compelling relationship between COMT polymorphisms and breast cancer carcinogenesis^70,71^. As the *MDR1* gene encodes the ATP-dependent cellular efflux pump p-glycoprotein, protecting cells from xenobiotics and metabolites, it has also had a proposed role in carcinogenesis. Multiple cancer types have been associated with the *MDR1 C3435T* polymorphism in a meta-analysis of case–control studies^72^. These findings suggest complex interrelationships between these genetic polymorphisms and carcinogenesis and may render individuals vulnerable to both cancer the neurotoxicity of its therapies. These relationships warrant further exploration.

In contrast to global connectivity, regional changes were found to be related to at-risk allele status. Regional connectivity involving left calcarine and left cuneus as well as right and left paracentral lobule was significantly lower in chemotherapy-treated patients treated with *BDNF* Met allele or *MDR1* T allele. Further, lower left calcarine and left cuneus connectivity was also associated with a higher number of at-risk alleles in the breast cancer population, independent of chemotherapy exposure, but not in healthy controls. These regions have had implicated vulnerability in other imaging studies of CRCI. In a study of hippocampal-cortical functional connectivity, increased functional connectivity was demonstrated between the hippocampus and left cuneus in breast cancer survivors, as opposed to healthy controls^73^. We previously observed significant hyper-activation in left (and right) cuneus among chemotherapy-treated breast cancer survivors compared to healthy controls during a verbal memory task^74^. Increased perfusion in the left cuneus and left calcarine has been demonstrated in patients with breast cancer exposed to chemotherapy compared to both healthy and chemotherapy-naïve controls, a finding also correlated with dysfunction changes in executive control network scores^75^. Our analysis suggests dysfunction in these regions may be influenced by the presence of at-risk alleles, a finding that warrants further exploration.

Another unique finding in our analysis were the differences in connectivity between left olfactory and left superior parietal lobe as well as left hippocampus and left rolandic operculum for healthy controls with the *APOE e4* allele. The olfactory system and hippocampi are of central interest in neurobiology given their roles in neurogenesis. Neurogenesis extends well-beyond the embryonic and post-natal system in select regions of the brain. The subventricular zone generates interneurons for the olfactory bulb, and the olfactory neurons generate new excitatory sensory neurons that send their axons to the olfactory bulb^76^. Similarly, adult neurogenesis also occurs in the subgranular zone of the hippocampal dentate gyrus^77^. Alterations of connectivity in these regions among individuals harboring the *APOE e4* allele may be associated with increased vulnerability to age-related cognitive decline. For example, *APOE e4* is a risk factor for Alzheimer’s disease, where neurogenesis is known to be dysfunctional^78^. However, post hoc analysis did not indicate significant differences in cognitive performance between healthy controls based on *APOE e4* genotype in this sample. It is also unclear why this finding was specific to healthy controls but may suggest an altered relationship between genotype and neurogenesis in patients with breast cancer that requires further study.

The potential for genetic polymorphisms to modulate the impact of neurologic insults has been identified in several diseases^79,80^, but is not defined in cancer. Recent systematic review has found variable correlations between genetic polymorphisms and CRCI^81^, precluding the conclusive definition of a relationship. Connectomics provides a novel contribution to elucidating this relationship. The data presented in this study supports at-risk alleles impacting vulnerability to CRCI, with the numbers of alleles expressed in an individual predicting poorer cerebral connectivity. Limitations of this study include the small number of patients studied and the differential frequencies of at-risk alleles compared to the normal population. The latency between initial breast cancer therapy and neurocognitive testing and imaging was greater than 5 years, significantly longer than most studies of CRCI, which generally evaluate patients within a year of treatment completion^82–84^. This prolonged interval may have influenced our identification of cognitive impairment on formal testing and our findings on connectome analysis. However, many studies have identified cognitive deficit in cross-sectional cohorts of breast cancer survivors who are decades off-therapy^85,86^. Therefore, many factors may contribute such as choice of neuropsychological tests and/or small sample. The focused study of breast cancer patients also suggests these findings should be validated in different oncology patient groups to determine their generalizability. Furthermore, a predictive relationship between these genetic polymorphisms and CRCI or connectome alterations cannot be definitively drawn given the lack of baseline neurocognitive and neuroimaging data. Larger prospective study in this area will allow for more definitive analysis of the influence of genetic polymorphisms on brain network topology and the development of CRCI. Ultimately, a comprehensive understanding of at-risk alleles to the development of cancer-related neurotoxicity may assist in identifying high-risk patients, allowing for appropriate counseling and risk-reduction strategies to be enacted where feasible.

### Research involving human participants and/or animals

All procedures performed in studies involving human participants were in accordance with the ethical standards of the institutional and/or national research committee (Stanford Institutional Review Board, #14623) and with the 1964 Helsinki declaration and its later amendments or comparable ethical standards.

### Consent to participate

Informed consent was obtained from all individual participants included in the study.

## Data Availability

The raw data supporting the conclusions of this manuscript will be made available by the authors, without undue reservation, to any qualified researcher.
